# Refining Scale Measurement: Reassessing Oral Impacts on Daily Performances Properties With Item Response Theory

**DOI:** 10.1111/cdoe.70034

**Published:** 2025-11-06

**Authors:** Roger Keller Celeste, Matheus de França Perazzo, Georgios Tsakos, Michael Reichenheim

**Affiliations:** ^1^ Department of Preventive and Social Dentistry Federal University of Rio Grande Do Sul Porto Alegre Brazil; ^2^ Aging Research Center Karolinska Institute Solna Sweden; ^3^ Faculty of Dentistry Federal University of Goiás Goiânia Brazil; ^4^ Faculty of Dentistry Evangelical University of Goiás Anápolis Brazil; ^5^ Department of Epidemiology and Public Health University College London London UK; ^6^ Institute of Social Medicine Hesio Cordeiro State University of Rio de Janeiro Rio de Janeiro Brazil

**Keywords:** factor analysis, item response theory, oral health‐related quality of life, patient‐reported outcome measures

## Abstract

**Objectives:**

Many oral health‐related quality of life instruments have been developed but few have undergone a comprehensive psychometric assessment. One commonly used measure is the Oral Impact on Daily Performance (OIDP). This study revised the configural and metric properties as well as the performance of items based on Item Response Theory (IRT) of a dichotomous‐item version of OIDP in Brazil.

**Methods:**

The nine‐item dichotomous version of the OIDP was analysed using data from a nationally representative sample from the Oral Health Survey (SBBrasil 2010). It consisted of 30 064 individuals aged 12 to 75 and was split into two partitions comprising n_1_ = 20 040 and n_2_ = 10 024, respectively. Confirmatory factor analyses (CFA) were conducted on the larger partition and cross‐validated on the smaller to assess configural and metric properties. The item performance was evaluated using a 2‐parameter item response theory (IRT) model. Sampling weights were used in all analyses.

**Results:**

The unidimensional model presented two violations of conditional independence, one between items i5 (practising sports) and i4 (going out) and another between items i6 (trouble in speaking) and i7 (shame of speaking or smiling). A CFA of the most parsimonious model (removing i5, i6 and i7) yielded a RMSEA = 0.02, WRMR = 1.42, CFI = 0.99 and TLI = 0.99. The IRT analyses showed that three pairs of items had very similar levels of difficulty and discrimination suggesting redundancy.

**Conclusions:**

A shorter dichotomous version of the OIDP scale has acceptable configural and metric properties. Being more concise and thus efficient, it may be better suited for large‐scale population surveys than the version currently in use.

## 1 Introduction

The relevance of Oral Health‐Related Quality of Life (OHRQoL) has long been established in oral health because tooth and mouth problems impact daily activities, disrupting work and study and having a high cost to individuals and society [1, 2]. Since the 1990s, research in this area has shown a steep and continuous growth [3]. In addition to the traditional generic OHRQoL instruments, more recent condition‐specific ones have been developed to tap this concept [4]. Nonetheless, the most used ones are short versions of generic scales, abridged to be applied in large epidemiological oral health surveys. Studies undertaken in several countries have supported construct validity and reliability as minimum psychometric properties for using some of the older generic instruments [5]. However, there are criticisms for using mostly external validation methods [4]. The current consensus highlights the importance of well‐conducted internal validation studies as the first stage [6], for example, using confirmatory factor analysis.

One of the oldest and most widely used instruments is the Oral Impacts on Daily Performances (OIDP), developed in 1996 [7, 8]. The instrument was developed concurrently in the UK and Thailand. It assesses the impact of oral health issues on daily activities by evaluating both the frequency and severity of these impacts. Each item's score is calculated by multiplying the frequency (on a 5‐point scale) by the severity (also on a 5‐point scale) of the impact caused by oral disorders on daily life. The scale consists of eight items probing three hypothesised theoretical dimensions: (a) physical performances (eating, speaking, cleaning); (b) psychological performances (sleeping, smiling, emotional state); and (c) social performances (working, contact with people) [7]. In the initial version, the authors included ‘light physical activities’ (e.g., sports and domestic work) as a ninth item in the physical performances dimension but removed it in a later version [8]. The nine‐item version has been translated and used in Brazil [9, 10] and it was included in the national oral health survey (SBBrasil) in a dichotomous item response version. Although the OIDP was developed for adults, it has also been used in several age groups; indeed, the Child‐OIDP [11, 12] has been applied to children over 11 years using the same items as the adult version [13]. The OIDP dimensionality and psychometric properties have been reported in countries such as Brazil, Peru, Norway, Spain, Sweden, Turkey, Madagascar, Tanzania, Uganda, Pakistan and Sri Lanka [12, 14, 15, 16, 17, 18, 19, 20, 21, 22, 23]. Overall, it has been translated to more than 80 countries in Africa, Asia, Europe, Latin America, and North America [4, 24].

Valid instruments require fine‐tuning. However, essential measurement properties are not always examined in depth, and this seems to be the case for many OHRQoL measures, including the OIDP [4]. The proposed configural 3‐factor model of OIDP has not been confirmed in studies, and there is reasonable evidence supporting one factor. First, violations of the original configural model have been reported, including a lack of item specificity to their respective dimension and item ambiguity, i.e., items loading into a different factor presenting unexpected cross‐loadings [12, 14, 15, 16, 17, 22]. Second, most studies described good reliability for the overall score, implying one dimension, with Cronbach's alpha values from 0.62 to 0.77. Third, each OIDP item is measured with a 25‐point scale to capture a fine grain of the construct, but it is possible that such long item scale captures some random error, explaining why a study using dichotomous items showed that the one‐factor solution had the best statistical fit [18] and with a ratio between the first and second eigenvalue higher than four, which strongly suggests unidimensionality [25]. Indeed, it has been suggested that using the OIDP frequency score could improve simplicity and efficiency [7]. Pilotto et al.'s study [18] also showed that the version using dichotomous items had good item reliabilities and no redundancies, presenting loadings above 0.50 and the absence of substantial residual correlations.

The state of the art of psychometric properties of OIDP indicates the need to review the configural and metric properties, given the changes in the Brazilian version. In spite of the mounting evidence from Confirmatory Factor Analyses (CFA), scalar properties of the unidimensional model have not been assessed so far. In fact, the instrument seems to adequately order items over the latent trait according to their impact levels of OHRQoL. For example, physical impacts are more frequently endorsed than those affecting social function—the item about avoiding contact with other people due to problems with mouth and teeth had only 2.4% of endorsement, while difficulties eating had 49.7% [7]. Items in a unidimensional scale are expected to discriminate the difficulty levels to avoid redundant items and to measure the intensity of the latent trait efficiently. Therefore, this study reviewed the configural and metric properties as well as the performance of items based on Item Response Theory (IRT) of a dichotomous‐item version of the OIDP in Brazil.

## 2 Methods

### 2.1 Design and Sample

This study used publicly available data from the Brazilian National Oral Health Survey 2010 (SBBrasil 2010), a nationally representative survey of the urban population. Data from four age groups were analysed: children 12 years old (*n* = 7328), adolescents 15–19 years old (*n* = 5445), adults 35–44 years old (*n* = 9779) and older individuals 65–74 years old (*n* = 7619). Amongst adults and older individuals, there were 2.2% and 1.3% exam refusals, respectively. However, due to the household sampling design, only 61% and 93% of the targeted sample size were reached for adults and older individuals, respectively, as households contained fewer adults than estimated. This study employed the complex sampling used in the survey, combining stratified and cluster sampling processes, and further details can be found elsewhere [26].

### 2.2 Instrument and the Item Map

The OIDP version applied in SB2010 has some differences from the original version; it included nine items (Table 1) in a standard questionnaire, administered via electronic devices during household interviews conducted by trained examiners. The questionnaire began with the introductory statement: ‘Some people have problems that can be caused by their teeth. Given the situations below, which applied to you in the last six months? Did (you) […]:’ followed by nine items: i1—[Did you] have difficulty eating because of your teeth or feel pain while drinking cold or hot beverages?; i2—Did your teeth bother you when brushing?; i3—Did your teeth make you nervous or irritated?; i4—Did you avoid going out, having fun, or attending parties because of your teeth?; i5—Did you stop doing sports because of your teeth?; i6—Did you have difficulty speaking because of your teeth?; i7—Did your teeth make you feel ashamed while smiling or speaking?; i8—Did your teeth cause trouble studying or working, or completing tasks at school or work?; i9—Did you stop sleeping or sleep poorly because of your teeth? The response to each question was either ‘yes’ (code 1) or ‘no’ (code 0).

**TABLE 1:** Item map of oral impact on daily performances.

Original hypothesised dimension	SBBrasil version items content	Original version items content	Frequency of endorsement
Physical performances	i1–Difficulty Eating	i1 – Eating	Less intense ↓ More Intense
	i2–Toothbrushing	i3 – Cleaning teeth	
	i6‐Trouble speaking	i2 – Speaking	
Psychological performances	i3‐Making nervous or irritated	i6 – Being irritable	
	i9‐Sleeping poorly	i4 – Sleeping and relaxing	
	i7‐Shame smiling or speaking	I5 – Smiling without embarrassment	
Social performances	i5‐Doing sport	i9 – Light Physical activities	
	i4‐Avoid going out	i7 – Contact with people (Leisure)	
	i8‐Trouble studying or working	i8 – Major work or social role	

The Wright Item Map [27] with the presumed order of intensity of impacts is described in Table 1, having some reference points (physical, psychological and social impact items). Everyday activities that individuals carry out on their own should be theoretically the first affected/refrained items because they can be avoided/altered/replaced without much impact. For example, this includes eating and cleaning one's teeth. On the other hand, activities that can hardly be avoided will be less affected/refrained. For example, carrying out major social roles such as going to school or work. The order of items endorsement within each dimension was obtained from a previous study [18].

### 2.3 Statistical Analysis

The sample was randomly split into two groups for CFA, with partition one being 2/3 of the total sample and partition two having the remaining 1/3. The unidimensional structure was evaluated in partition one and replicated in partition two. The initial model was a strict one, without residual correlations. The goodness‐of‐fit of the model to the data was evaluated using the following parameters: Comparative Fit Index (CFI), Tukey‐Lewis Index (TLI), Root Mean Square Error of Approximation (RMSEA), and Weighted Root Mean Square Residual (WRMR). The root mean square error of approximation (RMSEA) incorporates a penalty function for poor model parsimony, and values under 0.06 suggest close approximate (adequate) fit. The comparative fit index (CFI) and the Tucker‐Lewis index (TLI) represent incremental fit indices, and values > 0.95 are indicative of adequate fit [28], 70. WRMR values below or close to 1 indicate good fit; although it can increase with misspecification and sample size, it is mostly informative if the *p*‐value is not significant. The chi‐square test was used to compare the changes in χ^2^ between nested models (DIFFTEST command). A significant *p*‐value (*p* < 0.05) suggests that the imposed constraints were overly restrictive, and the constrained model should therefore be rejected.

The metric structure was evaluated assessing item loadings magnitude, coefficients sign (positive/negative), and residual correlations. High item loadings are expected, and they are related to items residuals, which are expected to be low. Residuals >|0.70| indicate that an item may be unreliable and may be considered for exclusion [29]. Potential reasons for the residual correlations were inspected individually and addressed in alternative models. Additional parameters were added and freely estimated if modification indexes were > 10 and the standardised magnitude of the coefficient was > 0.30.

Finally, the scaling properties were assessed separately in both partitions. A 2‐parameter probit item response theory (2PP IRT) was estimated to assess the items' difficulty (intensity) and discrimination (slope) abilities. Additionally, the item characteristic curve of each item was plotted in a graph. Items with close difficulty levels may be considered redundant, and all items are expected to have a similar high discrimination ability, and a formal t‐test (ttesti Stata command) of the difference between pairwise difficulty parameters was carried out using the standard errors provided by the software. CFAs were carried out in Mplus 7.11 and other analyses in Stata 19.

## 3 Results

The initial sample comprised 30 171 individuals, of which 107 (0.35%) had missing data for all OIDP items. The number of missing responses varied by item, ranging from 0.91% (*n* = 274 missing in item 5) to 0.51% (*n* = 155 missing in item 6). The weighted mean OIDP score was 1.35 (95% Confidence interval [95% CI]: 1.24–1.45), ranging from zero (56.9% of respondents) to nine (0.74% of respondents), with a median value equal to zero (interquartile range: 0–2). Table S1 shows that 58.3% of respondents were female, 44.8% had > 8 years of education, and the largest age group was adults 35–44 years old, with 40.3%. Most individuals were residents of the Southeast Brazilian region (59.8%). The item with the highest proportion of endorsement was i1 (around 25%, see Table S2) in any group by sex or age, while i5 was the item with the lowest proportion of endorsement (about 4%).

### 3.1 Assessment of Configural and Metric Structure

A comparison of fit indices of the initial unidimensional (1‐factor) model and different modified versions of that model is reported in Table 2 for the sample partition one (see Table S3 for the sample partition two). All models had an acceptable fit, despite violating item independence assumptions (items' residual error correlations). Two additional parameters were included and significantly improved the fit (Mplus chi‐square test (DIFFTEST) of nested models: initial vs. modified initial *p* < 0.01, Table 2).

**TABLE 2:** OIDP item factor loadings (standardised λ) and uniqueness (δ) 1‐factor models with and without modifications (larger sub‐sample *n* = 20 047).

F1 BY	Initial model		Modified initial model		Alternative Model 1		Alternative Model 2		Alternative Model 3		Alternative Model 4		Alternative Model 5	
	λ	δ	λ	δ	λ	δ	λ	δ	λ	δ	λ	δ	λ	δ
OIDP1	0.76	0.47	0.77	0.45	0.76	0.42	0.76	0.42	0.75	0.43	0.77	0.41	0.75	0.43
OIDP2	0.70	0.51	0.71	0.49	0.72	0.48	0.73	0.47	0.71	0.49	0.72	0.48	0.74	0.46
OIDP3	0.83	0.31	0.84	0.29	0.85	0.28	0.86	0.27	0.87	0.25	0.85	0.28	0.86	0.26
OIDP4	0.87	0.29	0.84	0.34	0.84	0.30	0.83	0.32	0.83	0.31	0.82	0.32	0.81	0.34
OIDP5	0.84	0.31	0.78	0.39										
OIDP6	0.84	0.29	0.81	0.37							0.81	0.34		
OIDP7	0.78	0.36	0.75	0.42					0.74	0.46				
OIDP8	0.84	0.30	0.85	0.27	0.85	0.28	0.83	0.31	0.87	0.25	0.83	0.31	0.83	0.32
OIDP9	0.81	0.34	0.82	0.32	0.81	0.34	0.83	0.32	0.80	0.36	0.83	0.31	0.84	0.30
OIDP6 and 7							0.81	0.35						
OIDP6 or 7					0.82	0.32								
**Residual Correlations**														
OIDP4 WITH OIDP5			0.52											
OIDP7 WITH OIDP6			0.41											
**Fit Indices:**														
CFI	0.98		0.99		0.99		0.99		0.99		0.99		0.99	
TLI	0.97		0.98		0.98		0.99		0.98		0.99		0.99	
RMSEA	0.02		0.01		0.02		0.02		0.02		0.02		0.02	
WRMR	1.89		1.44		1.61		1.38		1.68		1.41		1.42	

All factor loadings were > 0.70 and residuals below 0.50 (Tables 2 and S2 and Figures S2–S4). The initial unidimensional model presented two large residual correlations (> 0.30, *p* < 0.01). The first residual correlation was between items i4–i5 (contact with others during leisure time and physical activities/sports) and the second i6–i7 (trouble speaking and shame smiling or speaking). After detailed inspection, i5 was removed from all alternative models because it was deemed theoretically non‐universal; that is, not all individuals practice sports in general; hence they cannot stop doing it. This was also supported by the low endorsement of the item (only 4%). The other residual correlation may have been caused by the wording of items, given that both of them included the idea of ‘speaking problems,’ either related to shame or trouble. Five alternative models were further tested: (M1) a new item was generated merging individuals who endorsed either i6 or i7, (M2) a new item was generated merging individuals who endorsed both i6 and i7, (M3) removing i6, (M4) removing i7, and (M5) removing both i6 and i7. All alternative models presented very good metric properties, and the most parsimonious was model 5 (see Figures S2–S4).

### 3.2 Assessment of Scaling Structure

The order of intensity by endorsement of the items in Table 1 was confirmed only after removing three items (i5, i6 and i7), with i1 being the most common impact and i5 the least (Table S2). Merging i6 and i7, or alternatively using i6 or i7, proved to be redundant to other items tapping the same position in the latent trait (theta value in the x‐axis that shows the difficulty parameter). Indeed, in the 2‐parameter probit IRT (Tables S4 and S5), i6 was located in close position to i4 (not significant difference in difficulty level, *p*‐value = 0.63); likewise, i7 was located in a position close to i3 (not significant difference in difficulty level, *p*‐value = 0.41). The remaining 6 items (after removing i5, i6 and i7) spread almost evenly along the latent trait continuum ranging from about −1 to +4 of the horizontal axis (see Item Information Function [IIF], Figure S1), showing a lack of low‐intensity items, as most items were located in the positive part of the latent trait.

**FIGURE 1:**
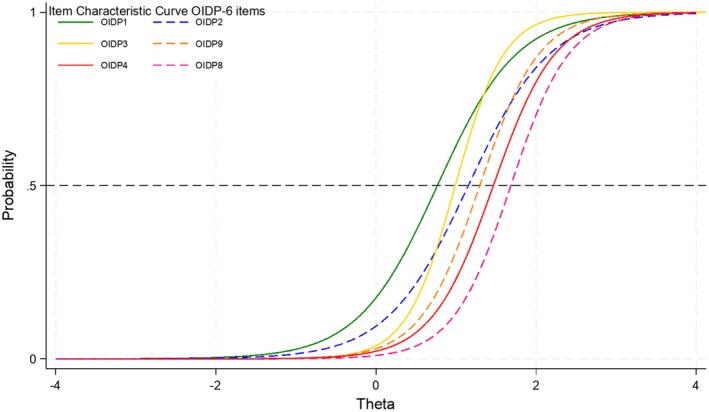
Item characteristic curve of a six‐item OIDP version (removing i5, i6 and i7).

Despite a good fit, the item characteristic curve showed that i3 (nervous or irritated) had a high discriminating slope, crossing i2 and i1 (Figure 1); while i2 (toothbrushing) had a low discriminating slope, crossing i3 and i9. Those situations violated an important assumption as items' order should not change over the continuum, unless it is located in the extreme upper or lower s‐shaped curves.

## 4 Discussion

The current study confirmed that a unidimensional model of a dichotomous scoring item version of the OIDP showed appropriate configural properties, as argued by Pilotto and colleagues [18]. This model also presented fewer violations in the metric evaluations, and alternative models were tested to deal with redundancy between items i4–i5 and i6–i7. Finally, an alternative model with six items performed better than the one with eight items, based on IRT results. This is the most comprehensive assessment of OIDP to the authors' knowledge.

This study has some important limitations. Validation is context‐dependent; therefore, the current findings may not be generalisable to other countries without proper cross‐cultural validation. This issue is sensitive because OIDP has several versions with different item content without a rigorous psychometric assessment [4]. The Brazilian version has a couple of items with different content from the original, and a change to a binary response alternative (yes/no) in each item. This new item‐scoring system needs further investigation. On one side, dichotomous items might lose the ability to discriminate different levels of the intensity of quality of life; on the other side, if individuals are not able to discriminate small degrees of intensity, a dichotomous response may reduce random error [18, 30].

The initial and alternative models in the current study provided evidence for a unidimensional structure. This finding confirmed another Brazilian study that assessed the structural dimension in a CFA, showing low discriminant validity of a three‐factor model with high correlations (*r* > 0.90) between factors [18]. Two previous studies reported moderate factor correlations when testing a multidimensional structure in Spain [17] and Madagascar [20], perhaps due to a different item‐scoring system (frequency times severity). Violations of the initial model also include conditional dependency between items. In this regard, two studies [16, 17] reported residual correlations between pairs of items different from the current work and no clear conclusion may be drawn. In the current study, there was a content overlap of items i6 and i7 tapping on speaking difficulties, both thus requiring revision. It is worth noting that the wording in item 7 is a feature of the OIDP version used in SBBrasil, referring to ‘shame’ that can be linked to ‘smiling’ and ‘speaking,’ but not in the original OIDP where the respective item refers to ‘embarrassment with smiling’. Moreover, item 5 was (re)introduced in the SBBrasil version based on an older preliminary version of the original OIDP, but ‘stop doing sports’ may not be conceptually universal (not everyone practices sports); hence, we opted to remove it. The low proportion of endorsement (around 5% in all age groups) suggests that people either do not practice sports or they did not stop doing it because of the impact of teeth and mouth problems. Although the original item ‘light physical activity’ can be universally applicable, its psychometric performance cannot be anticipated and should be evaluated in future studies.

The scaling structure assessment suggests that the OIDP items can be ordered along a latent trait, though mostly with items that tap theta values higher than zero. The somewhat low proportion of endorsement (43.1% of respondents with a zero score in all items) in many items can be explained because surveys include a high proportion of healthy participants, while most instruments, such as the OIDP, have been developed to measure impacts amongst individual affected by oral diseases. The most endorsed item was ‘difficulty in eating’ (item 1), which has been reported as the most endorsed item in other studies [8, 12, 14, 20, 23]. Although this is the first study to assess OIDP using IRT, the order of endorsement has been similarly published elsewhere [7, 12, 14, 20, 23, 31]. It is important to highlight that raw scores and scores obtained from factor analysis are strongly related, they are different and further analysis for scalar properties must be pursued with non‐parametric methods. In the current study, a 2‐parameter IRT identified a few issues (after removal of i5), most notably, the theta values (difficulty coefficient) of i6 and i7 were close to i4 and i2, respectively, and this suggests redundancy. This could be due to a differential item functioning, given that people with fewer teeth (mostly older adults) may have less trouble cleaning their teeth than fully dentate ones (younger). However, this hypothesis should be further explored in future studies investigating invariance of item functioning.

In conclusion, this study indicated that a dichotomous scoring item version of the OIDP without redundant items has acceptable configural, metric, and scaling properties, and would be much easier to use in large population surveys. The use of dichotomous items may be easier in practice, and a shorter unidimensional instrument can be logistically better because it demands less cognitive effort from respondents and facilitates data collection [18, 30]. Further studies are warranted to assess whether the same items could or should be removed based on redundancy. It is important to confirm this in different contexts/cultures before implementing a final modification. Additionally, it is suggested to examine item functioning and effect invariance (using IRT), for instance across age groups.

## Author Contributions

R.K.C., M.F.P., G.T. and M.R. conceptualised the manuscript. R.K.C. carried out statistical analysis and wrote a draft of the manuscript. All authors interpreted results and revised critically the manuscript. All authors approved final version.

## Ethics Statement

This study was performed in line with the principles of the Declaration of Helsinki. The SBBrasil 2010 Survey was approved by the National Research Ethics Committee (number 15498).

## Consent

Written informed consent was obtained from study participants.

## Conflicts of Interest

The authors declare no conflicts of interest.

## Supporting information


**Data S1:** cdoe70034‐sup‐0001‐TableS1‐S5‐FigureS1‐S4.docx.

## Data Availability

The data that support the findings of this study are available in Ministério da Saúde do Brasil at https://www.gov.br/saude/pt‐br/composicao/saps/brasil‐sorridente/sb‐brasil/dados. These data were derived from the following resources available in the public domain:—Ministério da Saúde—Projeto SBBrasil, https://www.gov.br/saude/pt‐br/composicao/saps/brasil‐sorridente/sb‐brasil/dados.
