# Supplementary material for: Refining Scale Measurement: Reassessing Oral Impacts on Daily Performances Properties With Item Response Theory
Source: Community Dent Oral Epidemiol. 2025 Nov 6;54(1):126–32. doi: 10.1111/cdoe.70034 (PMC12808854; doi:10.1111/cdoe.70034)
Supplement: Supplementary file 1 — Data S1: cdoe70034‐sup‐0001‐TableS1‐S5‐FigureS1‐S4.docx. [file CDOE-54-126-s001.docx]

**Supplementary Tables** **and Figures**

**Table S1** – Weighted means OIDP score by sociodemographic characteristic of the sample.

|  |  | Weighted % | N | Weighted  OIDP score | (95%CI) |
| --- | --- | --- | --- | --- | --- |
| Sex | Female | 58.3 | 17758 | 1.50 | (1.38-1.62) |
|  | Male | 41.7 | 12413 | 1.13 | (1.01-1.25) |
| Age group | 12-year-old | 7.9 | 7328 | 0.86 | (0.72-1.00) |
|  | 15-19-year-old | 31.0 | 5445 | 0.99 | (0.86-1.12) |
|  | 35-44-year-old | 40.3 | 9779 | 1.78 | (1.58-1.97) |
|  | 65-74-year-old | 20.7 | 7619 | 1.23 | (1.10-1.36) |
| Education | >8 years | 44.8 | 10738 | 1.09 | (0.97-1.22) |
|  | 5-8 year | 31.6 | 11908 | 1.44 | (1.30-1.58) |
|  | <4 years | 23.5 | 7200 | 1.70 | (1.53-1.86) |
| Region | North | 7.3 | 7453 | 1.30 | (1.16-1.44) |
|  | Northeast | 10.5 | 8229 | 1.19 | (1.08-1.30) |
|  | Southeast | 59.8 | 5150 | 1.42 | (1.25-1.59) |
|  | South | 15.7 | 4629 | 1.18 | (1.05-1.32) |
|  | Midwest | 6.7 | 4710 | 1.36 | (1.21-1.51) |

**Table S2** - Proportion of endorsement of OIDP items by age group and sex.

|  | Overall sample (n=29482) | Women (n=17300) | Men (n=12182) | Adults and Older Individuals (n=16879) | Children and Adolescents (n=12603) |
| --- | --- | --- | --- | --- | --- |
| Item | Endorsement | Endorsement | Endorsement | Endorsement | Endorsement |
| oidp1 | 25.2% | 26.7% | 21.7% | 30.1% | 17.4% |
| oidp7 | 18.4% | 20.2% | 14.8% | 21.6% | 13.1% |
| oidp2 | 16.2% | 17.9% | 13.0% | 18.1% | 12.9% |
| oidp3 | 15.1% | 16.9% | 11.6% | 17.5% | 11.0% |
| oidp9 | 11.1% | 11.8% | 9.7% | 12.1% | 9.3% |
| oidp6 | 10.5% | 11.1% | 8.8% | 13.9% | 5.0% |
| oidp4 | 9.3% | 10.0% | 7.6% | 11.1% | 6.2% |
| oidp8 | 6.8% | 7.1% | 5.8% | 7.7% | 4.9% |
| oidp5 | 4.5% | 4.3% | 4.4% | 4.6% | 4.1% |
| No item endorsed | 56.9% | 53.9% | 61.1% | 51.7% | 63.9% |

**Table S3** – OIDP item factor loadings (standardised λ) and uniqueness (δ) 1-factor models with and without modifications (partition two n=10024)

|  | **Initial Model** | | **Modified Initial Model** | | **Alternative Model 1** | | **Alternative Model 2** | | **Alternative Model 3** | | **Alternative Model 4** | | **Alternative Model 5** | |
| --- | --- | --- | --- | --- | --- | --- | --- | --- | --- | --- | --- | --- | --- | --- |
| **F1 BY** | λ | δ | λ | δ | λ | δ | λ | δ | λ | δ | λ | δ | λ | δ |
| **OIDP1** | ***0.76*** | 0.43 | ***0.77*** | 0.41 | **0.75** | 0.44 | **0.73** | 0.47 | **0.75** | 0.43 | **0.74** | 0.45 | **0.73** | 0.46 |
| **OIDP2** | ***0.69*** | 0.52 | ***0.70*** | 0.51 | **0.73** | 0.47 | **0.73** | 0.47 | **0.75** | 0.44 | **0.72** | 0.48 | **0.72** | 0.48 |
| **OIDP3** | ***0.83*** | 0.31 | ***0.84*** | 0.29 | **0.85** | 0.28 | **0.85** | 0.27 | **0.84** | 0.30 | **0.85** | 0.27 | **0.86** | 0.25 |
| **OIDP4** | ***0.91*** | 0.17 | ***0.87*** | 0.24 | **0.83** | 0.31 | **0.83** | 0.31 | **0.82** | 0.32 | **0.83** | 0.31 | **0.83** | 0.31 |
| **OIDP5** | ***0.87*** | 0.24 | ***0.81*** | 0.34 |  |  |  |  |  |  |  |  |  |  |
| **OIDP6** | ***0.79*** | 0.37 | ***0.76*** | 0.43 |  |  |  |  |  |  | **0.72** | 0.49 |  |  |
| **OIDP7** | ***0.75*** | 0.44 | ***0.71*** | 0.49 |  |  |  |  | **0.77** | 0.41 |  |  |  |  |
| **OIDP8** | ***0.86*** | 0.26 | ***0.88*** | 0.23 | **0.85** | 0.28 | **0.85** | 0.27 | **0.83** | 0.32 | **0.85** | 0.28 | **0.83** | 0.31 |
| **OIDP9** | ***0.78*** | 0.39 | ***0.80*** | 0.37 | **0.81** | 0.35 | **0.80** | 0.36 | **0.82** | 0.33 | **0.81** | 0.35 | **0.81** | 0.35 |
| **OIDP6 and 7** |  |  |  |  |  |  | **0.77** | 0.41 |  |  |  |  |  |  |
| **OIDP6 or 7** |  |  |  |  | **0.74** | 0.45 |  |  |  |  |  |  |  |  |
| **Residual Correlations** | | | | | | | | | | | | | | |
| **OIDP4 WITH OIDP5** |  |  | ***0.66*** |  |  |  |  |  |  |  |  |  |  |  |
| **OIDP7 WITH OIDP6** |  |  | ***0.38*** |  |  |  |  |  |  |  |  |  |  |  |
| **Fit Indices:** |  |  |  |  |  |  |  |  |  |  |  |  |  |  |
| **CFI** | 0.99 |  | 1.00 |  | 1.00 |  | 1.00 |  | 0.99 |  | 1.00 |  | 1.00 |  |
| **TLI** | 0.98 |  | 0.99 |  | 1.00 |  | 1.00 |  | 0.99 |  | 1.00 |  | 0.99 |  |
| **RMSEA** | 0.02 |  | 0.01 |  | 0.01 |  | 0.01 |  | 0.01 |  | 0.01 |  | 0.01 |  |
| **WRMR** | 1.31 |  | 0.85 |  | 0.92 |  | 0.93 |  | 0.94 |  | 0.88 |  | 0.91 |  |

**Table S4 –** Difficulty (theta) and discrimination (slope) coefficients with standard errors **(**Std.Er.) from probit model Item Response Theory (partition two n=10024).

|  | 7 items (removing i6) | | 7 items (removing i7) | | 7 items (merging i7 and i6) | | 7 items (merging i7 or i6) | | 6 items (removing i7 and i6) | |
| --- | --- | --- | --- | --- | --- | --- | --- | --- | --- | --- |
|  | Difficulty | | | | | | | | | |
| Item | Coeff | Std. Err. | Coeff | Std. Err. | Coeff | Std. Err. | Coeff | Std. Err. | Coeff | Std. Err. |
| oidp1 | 0.78 | 0.07 | 0.77 | 0.06 | 0.78 | 0.07 | 0.77 | 0.06 | 0.78 | 0.07 |
| oidp6or7 |  |  |  |  |  |  | 1.07 | 0.06 |  |  |
| oidp3 | 1.08 | 0.07 | 1.08 | 0.07 | 1.08 | 0.07 | 1.08 | 0.07 | 1.06 | 0.07 |
| oidp7 | 1.25 | 0.07 |  |  |  |  |  |  |  |  |
| oidp2 | 1.20 | 0.08 | 1.22 | 0.08 | 1.21 | 0.08 | 1.21 | 0.08 | 1.22 | 0.08 |
| oidp9 | 1.44 | 0.07 | 1.43 | 0.07 | 1.44 | 0.07 | 1.43 | 0.07 | 1.43 | 0.07 |
| oidp4 | 1.49 | 0.07 | 1.50 | 0.07 | 1.49 | 0.07 | 1.50 | 0.07 | 1.49 | 0.08 |
| oidp6 |  |  | 1.74 | 0.10 |  |  |  |  |  |  |
| oidp8 | 1.76 | 0.09 | 1.76 | 0.10 | 1.75 | 0.10 | 1.77 | 0.09 | 1.80 | 0.10 |
| oidp6and7 |  |  |  |  | 1.86 | 0.09 |  |  |  |  |
| ttest p-value* | P=0.64 |  | P=0.89 |  | P=0.62 |  | P=0.91 |  |  |  |
|  | Discrimination | | | | | | | | | |
| Item | Coeff | Std. Err. | Coeff | Std. Err. | Coeff | Std. Err. | Coeff | Std. Err. | Coeff | Std. Err. |
| oidp1 | 1.09 | 0.09 | 1.10 | 0.09 | 1.07 | 0.09 | 1.12 | 0.09 | 1.08 | 0.09 |
| oidp6or7 |  |  |  |  |  |  | 1.10 | 0.10 |  |  |
| oidp3 | 1.60 | 0.12 | 1.63 | 0.13 | 1.63 | 0.13 | 1.60 | 0.12 | 1.71 | 0.15 |
| oidp7 | 1.06 | 0.09 |  |  |  |  |  |  |  |  |
| oidp2 | 1.08 | 0.09 | 1.05 | 0.09 | 1.07 | 0.09 | 1.07 | 0.09 | 1.05 | 0.09 |
| oidp9 | 1.35 | 0.13 | 1.36 | 0.13 | 1.34 | 0.13 | 1.36 | 0.13 | 1.38 | 0.13 |
| oidp4 | 1.50 | 0.13 | 1.49 | 0.13 | 1.50 | 0.13 | 1.48 | 0.13 | 1.50 | 0.13 |
| oidp6 |  |  | 1.03 | 0.09 |  |  |  |  |  |  |
| oidp8 | 1.62 | 0.18 | 1.60 | 0.18 | 1.64 | 0.19 | 1.59 | 0.17 | 1.49 | 0.17 |
| oidp6and7 |  |  |  |  | 1.20 | 0.11 |  |  |  |  |

**Table S5 –** Difficulty (theta) and discrimination (slope) coefficients with standard errors **(**Std.Er.) from probit model Item Response Theory (partition one).

|  | **7 items (removing i6)** | | **7 items (removing i7)** | | **7 items (merging i7 and i6)** | | **7 items (merging i7 or i6)** | | **6 items (removing i7 and i6)** | |
| --- | --- | --- | --- | --- | --- | --- | --- | --- | --- | --- |
|  | Difficulty | | | | | | | | | |
| Item | Coeff | Std. Err. | Coeff | Std. Err. | Coeff | Std. Err. | Coeff | Std. Err. | Coeff | Std. Err. |
| **oidp1** | 0.78 | 0.05 | 0.77 | 0.05 | 0.78 | 0.05 | 0.78 | 0.05 | 0.79 | 0.05 |
| **oidp6or7** |  |  |  |  |  |  | 0.84 | 0.04 |  |  |
| **oidp3** | 1.01 | 0.05 | 1.02 | 0.05 | 1.01 | 0.05 | 1.02 | 0.05 | 1.01 | 0.05 |
| **oidp7** | 1.07 | 0.05 |  |  |  |  |  |  |  |  |
| **oidp2** | 1.19 | 0.07 | 1.20 | 0.07 | 1.19 | 0.07 | 1.20 | 0.07 | 1.17 | 0.07 |
| **oidp9** | 1.32 | 0.06 | 1.29 | 0.06 | 1.29 | 0.06 | 1.32 | 0.06 | 1.28 | 0.06 |
| **oidp4** | 1.46 | 0.05 | 1.48 | 0.05 | 1.48 | 0.05 | 1.45 | 0.05 | 1.50 | 0.05 |
| **oidp6** |  |  | 1.44 | 0.06 |  |  |  |  |  |  |
| **oidp8** | 1.67 | 0.07 | 1.69 | 0.07 | 1.69 | 0.07 | 1.66 | 0.06 | 1.70 | 0.07 |
| **oidp6and7** |  |  |  |  | 1.69 | 0.07 |  |  |  |  |
| ttest p-value* | P=0.41 |  | P=0.63 |  | P=0.97 |  | P=0.39 |  |  |  |
|  | Discrimination | | | | | | | | | |
| Item | Coeff | Std. Err. | Coeff | Std. Err. | Coeff | Std. Err. | Coeff | Std. Err. | Coeff | Std. Err. |
| **oidp1** | 1.17 | 0.08 | 1.19 | 0.08 | 1.18 | 0.08 | 1.18 | 0.07 | 1.14 | 0.07 |
| **oidp6or7** |  |  |  |  |  |  | 1.45 | 0.09 |  |  |
| **oidp3** | 1.64 | 0.11 | 1.62 | 0.11 | 1.65 | 0.12 | 1.61 | 0.11 | 1.69 | 0.12 |
| **oidp7** | 1.17 | 0.06 |  |  |  |  |  |  |  |  |
| **oidp2** | 1.06 | 0.07 | 1.04 | 0.07 | 1.05 | 0.07 | 1.04 | 0.07 | 1.09 | 0.07 |
| **oidp9** | 1.36 | 0.08 | 1.49 | 0.09 | 1.46 | 0.09 | 1.38 | 0.08 | 1.52 | 0.10 |
| **oidp4** | 1.53 | 0.11 | 1.45 | 0.11 | 1.46 | 0.11 | 1.54 | 0.11 | 1.39 | 0.10 |
| **oidp6** |  |  | 1.38 | 0.09 |  |  |  |  |  |  |
| **oidp8** | 1.57 | 0.13 | 1.49 | 0.13 | 1.49 | 0.13 | 1.60 | 0.13 | 1.47 | 0.12 |
| **oidp6and7** |  |  |  |  | 1.37 | 0.10 |  |  |  |  |

* t-test comparing coefficients underlined


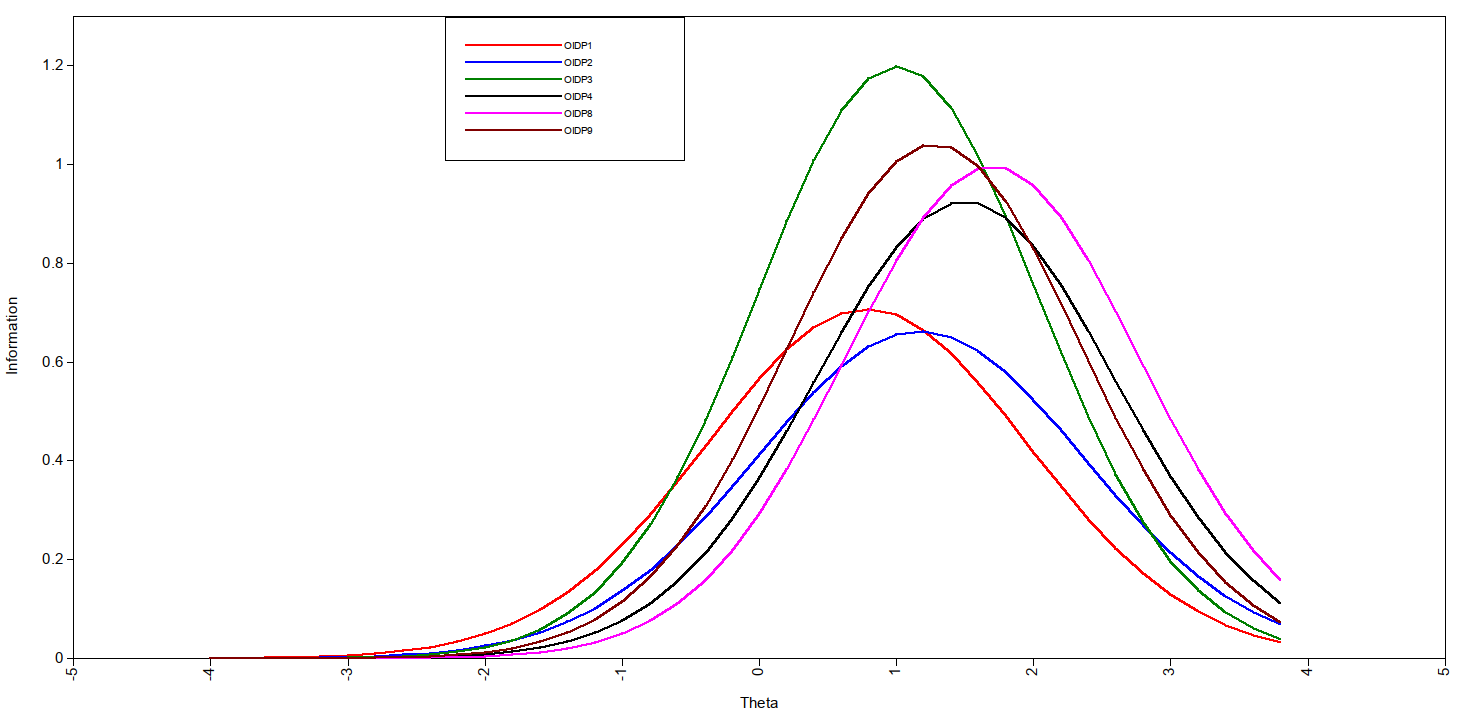


**Figure S1** – Item Information Function from 2 parameters item response theory of 6-item OIDP version in Brazil


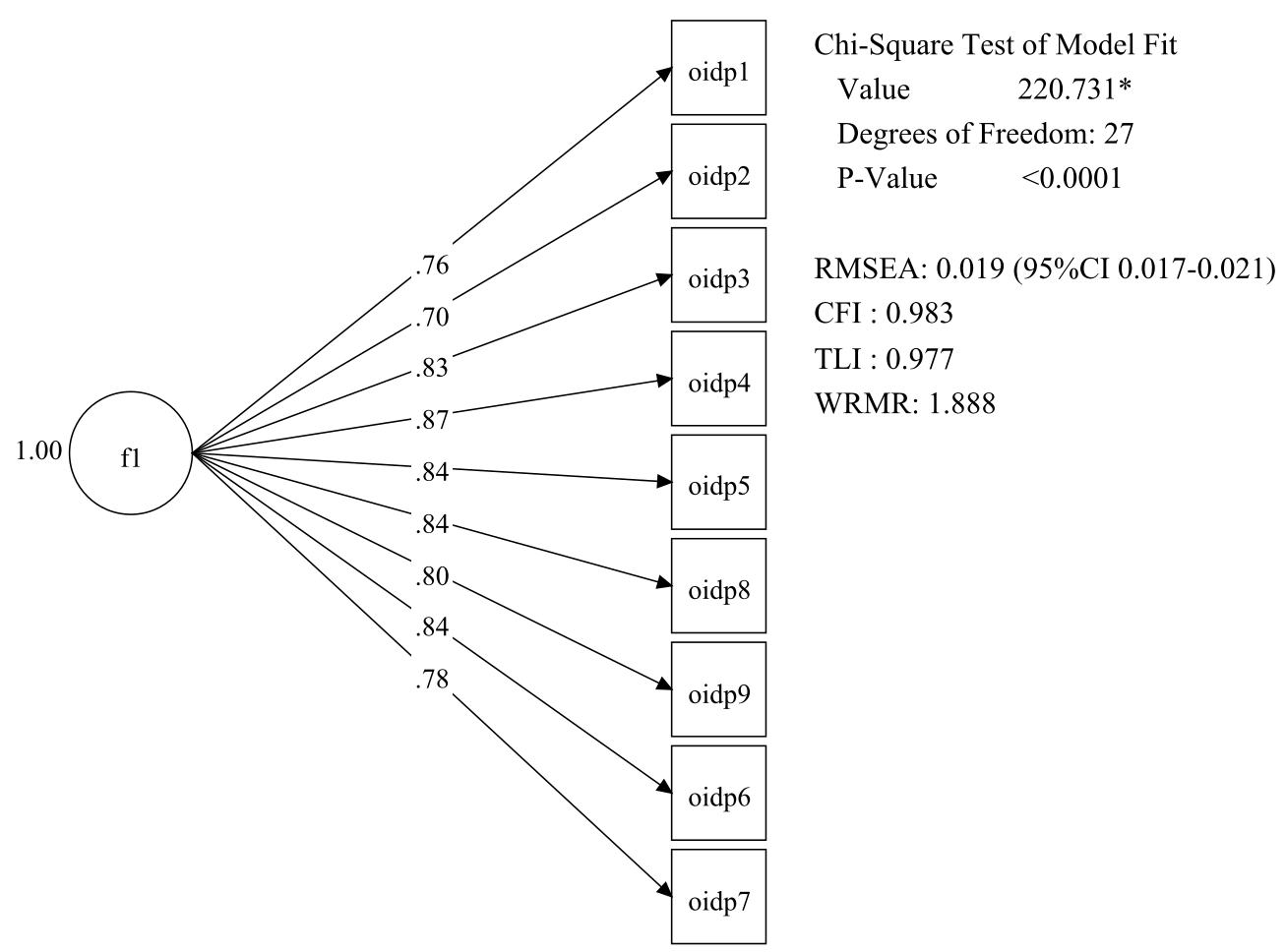


**Figure S2 -** Original OIDP model with item factor loadings (standardised λ) and fit indices from 1-factor models (partition one n=10024).


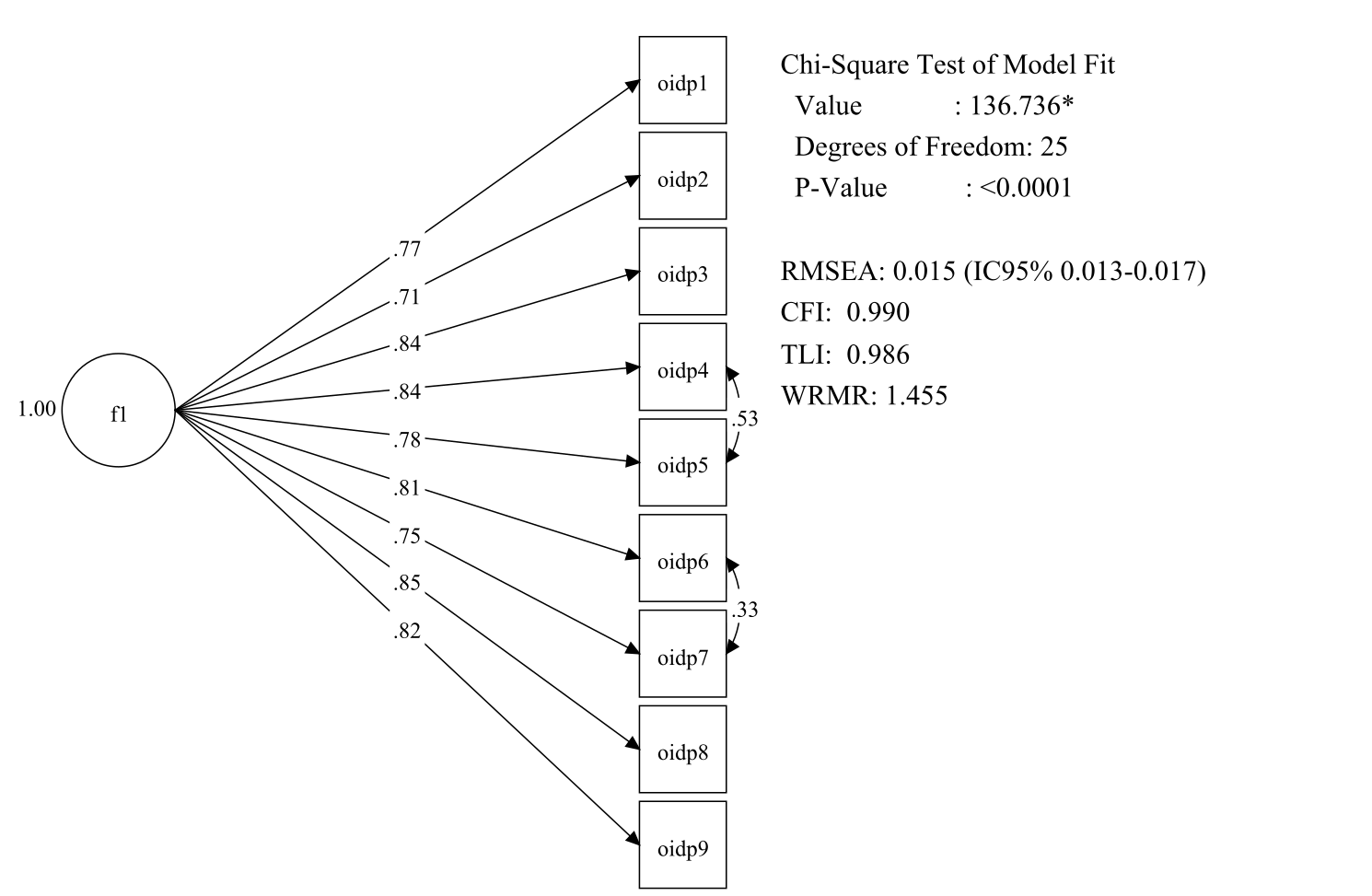


**Figure S3 -** Alternative OIDP model with item factor loadings (standardised λ), significant residual correlations and fit indices from 1-factor models (partition one n=10024).


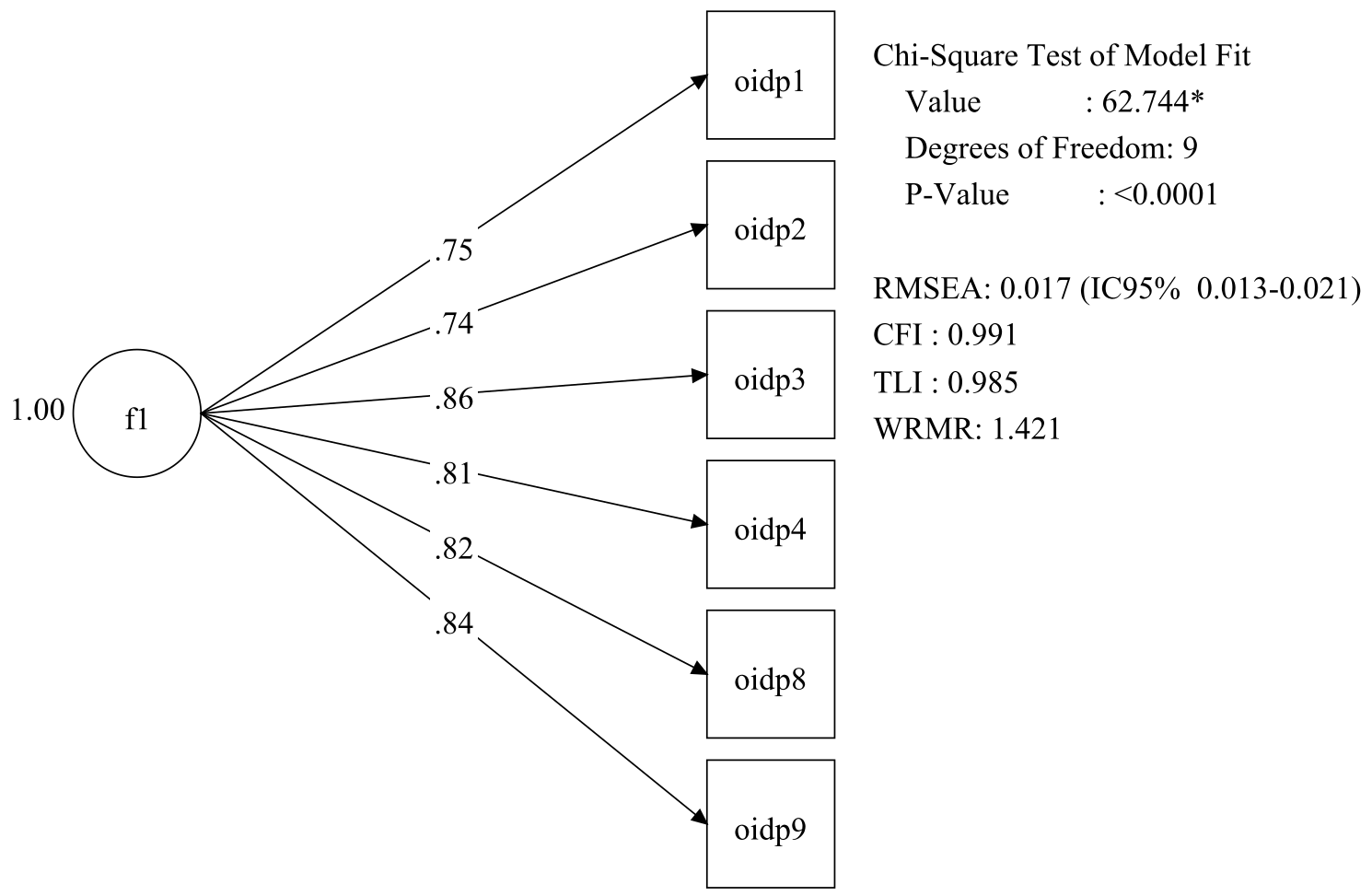
**Figure S4 –** Alternative OIDP model (Model 5: removing 3 items, no residual correlation) with item factor loadings (standardised λ) and fit indices from 1-factor models (partition one n=10024).
